# Comparative study of four innovative earth-friendly platforms for rapid analysis of daclatasvir dihydrochloride: Application on different matrices

**DOI:** 10.1186/s13065-023-00923-4

**Published:** 2023-03-15

**Authors:** Rania A. Sayed, Ahmed R. Mohamed, Wafaa S. Hassan, Manal S. Elmasry

**Affiliations:** 1 Analytical Chemistry Department, Faculty of Pharmacy, Zagazig University, Zagazig, 44519 Egypt; 2 Analytical Chemistry Department, Faculty of Pharmacy, Egyptian Russian University, Badr City, Cairo 11829 Egypt

**Keywords:** Daclatasvir dihydrochloride, COVID-19, Silver-nanoparticles, Electrochemical

## Abstract

**Background:**

Daclatasvir dihydrochloride has important roles not only in the management of COVID-19 pandemic symptoms but also in the treatment of chronic hepatitis C infection.

**Objective:**

The current research presents four novel and simple platforms including silver-nanoparticles spectrophotometric technique and three electrochemical conductometric ones for daclatasvir analysis in its tablet, biological fluids, and dissolution media.

**Methods:**

The spectrophotometric platform involved the synthesis of silvernanoparticles through a redox reaction between the reducing agent (daclatasvir) and the oxidizing agent (silver nitrate) in presence of polyvinylpyrrolidone as a stabilizing agent. The produced silver-nanoparticles have an intense surface plasmon resonance peak at 421 nm where the measured absorbance values were utilized for quantitative spectrophotometric determination of daclatasvir. While the electrochemical conductometric platforms involved the reaction of daclatasvir with three different precipitating reagents (silver nitrate, phosphomolybdic acid, and ammonium reineckate) to form ion associates between these reagents and daclatasvir in the aqueous system.

**Results:**

All proposed platforms were validated in line with recommendations of the international conference on harmonization producing satisfactory outcomes within the agreed boundaries.

**Conclusion:**

The proposed platforms are green alternatives for routine rapid assay of daclatasvir at the cheapest cost because their results were observed to be nearly similar to those of the reported platform. Moreover, the suggested spectrophotometric platform’s sensitivity can be employed for investigating daclatasvir bioequivalence.

**Supplementary Information:**

The online version contains supplementary material available at 10.1186/s13065-023-00923-4.

## Introduction

**Fig. 1:**
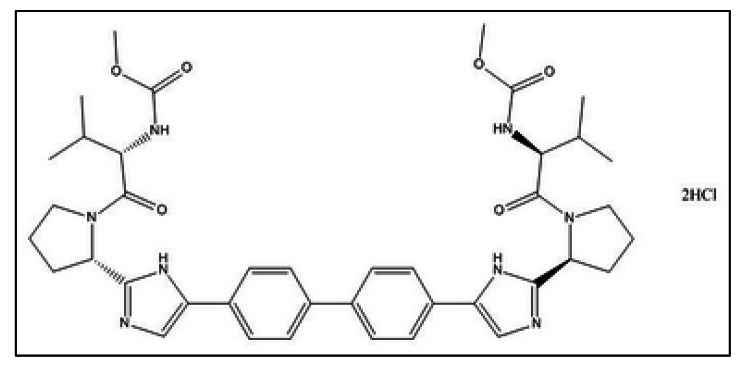
Chemical structure of daclatasvir dihydrochloride

Daclatasvir dihydrochloride (DACH) presented in Fig. 1, is a first-in-class direct-acting antiviral agent which is efficient against chronic hepatitis C virus (HCV) infection that is the leading cause of many deaths globally due to cirrhosis and hepatocellular carcinoma. DACH is potent against all HCV genotypes [1–6] due to its ability to impede the function of HCV protein (NS5A) which is necessary for HCV replication [1, 2]. Furthermore, DACH has a prodigious role in the management and treatment of patients with moderate or severe COVID-19 symptoms via targeting the early events during the replication cycle of SARS-CoV-2 (the main contributor to the COVID-19 pandemic) and preventing the induction of TNF-α, IL-6, and inflammatory mediators correlated with the cytokine storm of SARS-CoV-2 infectious disease [3–5]. Consequently, DACH improves the clinical rates of recovery and decreases the length of stay in the hospital.

Silver-nanoparticles (Ag-NPs) exhibit broad-ranging antimicrobial actions in addition to their immense roles in various fields, particularly those relating to drug delivery (DD) and/or drug analysis (DA). In the arena of DD, Ag-NPs are employed to guide medications to sickly tissues accordingly, bettering the curative efficiency & lessening the probable drugs’ adverse influences, especially in chemotherapy arena [6]. In DA arena, Ag-NPs are employed to create numerous delicate & eco-friendly platforms for quantitative assay of medications at the cheapest price [7]. Also, Ag-NPs as a technique (method) of analysis can determine the analyzed drugs in nano-range and therefore this advantage was exploited to enhance the proposed spectrophotometric platform’s sensitivity for DACH determination in different biological fluids.

Electrochemical conductometric techniques are regarded to be one of the most efficient analytical platforms utilized in medication standardization. The titrations that utilize silver nitrate (AgNO_3_) or phosphomolybdic acid (PMA) or ammonium reineckate (Amm.Rt) as precipitating reagents have numerous advantages such as simplicity, time-saving, high accuracy results, and low analysis costs. According to the aforementioned advantages, these platforms are commonly utilized for rapid green analysis of many pharmaceuticals at the cheapest cost [8, 9].

According to the literature survey, it was found that some platforms were reported for estimation of DACH either alone or in combination with other antivirals in the pharmaceutical formulations or biological fluids involving spectrophotometric [10, 11], spectrofluorimetric [12, 13], electrochemical [14–16], and chromatographic platforms [17–28]. Hence, the target of this study is to present innovative, simple, economic, and time-saving platforms including spectrophotometric Ag-NPs technique and three electrochemical conductometric ones using three precipitating reagents (AgNO_3_, PMA, and Amm.Rt) for DACH rapid assay in its market formulation, human biological fluids, and dissolution media without any interference by any matrix producing excellent outcomes relative to previous technique [11]. Also, they are considered eco-friendly alternatives for rapid DACH assay in its pure form and pharmaceutical formulation due to their reliance on water as an inexpensive and ecological solvent comparable to the other high-priced & perilous solvents employed in most previous (reported) platforms. Additionally, the Ag-NPs platform’s sensitivity can be employed for investigating DACH pharmacokinetics in human fluids.

## Experimental

### Chemicals

All chemicals or reagents consumed during this research were of analytical grade; the bi-distilled water (BDW) was utilized during the entire study.

- Standard of DACH (99.21%) was supplied from Mash Premiere (New Cairo, Egypt).
- Methanol, acetone, and ethanol (Adwic, Egypt).
- NaOH, (5 × 10^− 3^ M) aqueous solution (Adwic, Egypt).
- AgNO_3_, (3 × 10^− 3^ M and 5 × 10^− 3^ M) aqueous solutions (Sigma-Aldrich, Egypt), should be prepared freshly and shielded from light during usage.
- Polyvinylpyrrolidone (PVP), (0.14%) aqueous solution (Sigma-Aldrich, Egypt).
- Phosphomolybdic acid and ammonium reineckate, (5 × 10^− 3^ M) aqueous solutions (Sigma-Aldrich, Egypt).
- Potassium dihydrogen orthophosphate, (5 × 10^− 2^ M) aqueous solution (Oxford, India).

—Dissolve 20.4 gm of potassium dihydrogen orthophosphate in three liters of BDW, then pH of the solution was adjusted using analytical grade orthophosphoric acid (Biotech, Egypt) (to prepare phosphate buffer pH = 6.8).

- Human plasma samples were provided from Zagazig Hospitals (Egypt) & at − 20 °C were preserved pending the analysis time.
- Human urine samples were collected from healthy volunteers and kept frozen until the analysis time.

### Pharmaceutical formulation

Daklanork® tablets; manufactured by Mash Premiere, batch number (M171015), labeled to contain 66 mg DACH per tablet.

### Instruments

Jasco V-630 double-beam (Japan) UV-visible spectrophotometer equipped with two quartz cells (1-cm), connected to a DELL computer with spectra manager-2 software was exploited for measuring the absorbance values over the range (200–800 nm).

Jenway V-470 portable conductivity/TDS/Meter with a K_cell_ (cell constant) of 1.09 cm^− 1^ was utilized for measuring the conductance values.

A JEOL-1010 transmission-electron microscope at 80 kV (Japan) was employed for transmission electron microscopy (TEM) examination at Al-Azhar University.

USP dissolution type-II (Paddle) apparatus (V. VanKel VK 7000) was utilized for in-vitro dissolution testing. Also, sonicator (V. WUC-A06H), vortex mixer (V. VM-300), benchtop centrifuge (V. K241R), and pH-meter (V. Jenway-3510) were utilized.

### Standard solutions

For conductometric platforms processing, a standard DACH working solution (1 mg/mL) was prepared via dissolving 250 mg of pure DACH in 150 mL BDW as a primarily green solvent into a 250-mL volumetric flask (VF) for 15 min using the sonicator. Then, the volume was totaled using BDW to 250 mL.

For Ag-NPs platform processing, a standard DACH working solution (20 µg/mL) was prepared via dissolving 5 mg of pure DACH in 200 mL BDW into a 250-mL VF for 5 min using the sonicator. Then, the volume was totaled using BDW to 250 mL. The standard solutions were assessed to be stable for 14 days when kept in the fridge as they demonstrated no chromatographic changes, not even absorbance changes.

## General procedures

### Ag-NPs platform’s procedure

By utilizing a micropipette, volumes from DACH standard solution (20 µg/mL) were transferred accurately and followed by adding AgNO_3_ (1 mL, 3 × 10^− 3^ M), PVP (1.2 mL, 0.14%), and NaOH (1 mL, 5 × 10^− 3^ M) solutions into a group of 10-mL VFs. Then, the volumes were totaled via BDW to 10-mL marks to prepare solutions from 0.5 to 5 µg/mL. The prepared concentrated solutions were warmed for 30 min in the water bath (90 °C). After cooling to ambient temperature, the absorbance values were measured 3 times (for each solution) at 421 nm against the blank treated similarly without DACH.

### Electrochemical conductometric platforms’ procedure

To a series of 50-mL VFs, aliquots from DACH standard solution (1 mg/mL) containing (2–14 mg) were transferred. Then, the aliquots were totaled via BDW to the 50-mL mark. The contents of each VF were transferred to a glass beaker and then, the conductivity cell was submersed in the sample solution that was titrated against (5 × 10^− 3^ M) solutions of AgNO_3_, PMA, and Amm.Rt. For each addition of the proposed precipitating reagents and after every two minutes of thorough stirring, the conductance values were recorded and subsequently corrected for dilution influences according to the represented Eq. (1) [9], supposing that the relationship between dilution and conductivity (conductance) is linear.

1 $${\Omega ^{ - 1}}correct = {\Omega ^{ - 1}}obs\left[ {\frac{{{V_1} + {V_2}}}{{{V_1}}}} \right]$$

Where:

Ω^−1^_correct_ indicates the corrected electrolytic conductance; Ω^−1^_obs_ indicates the observed electrolytic conductance; V_1_ indicates the sample’s initial volume; V_2_ indicates the added volume of the proposed precipitating reagent.

The corrected conductivity was plotted against the added volume of the proposed reagent and consequently, the endpoint was graphically computed at the two lines’ intersection point. By using the graphically resulted endpoint in the represented Eq. (2) [9], the amount of DACH was computed easily as follows:

2 $$AmountofDACH(mg) = \frac{{V.M.R}}{N}$$

Where:

V indicates the volume of the proposed reagent; M indicates the molecular weight of DACH; R indicates the molarity of the proposed reagent; N indicates the number of moles of the proposed reagent consumed/one mole of DACH.

### Molar ratio procedure

The molar ratio of the conductometric platforms was performed by preparing equimolar solutions (5 × 10^− 3^ M) of DACH and each proposed reagent (AgNO_3_, PMA, and Amm.Rt) using BDW. Subsequently, certain volumes of DACH (5 × 10^− 3^ M) solution were accurately transferred into 50-mL VFs and treated as represented before under the electrochemical conductometric platforms’ general procedure for computing the DACH-reagent molar ratio for each proposed reagent.

### Application to the pharmaceutical formulation

Six tablets of Daklanork® were weighed, finely pulverized, and homogeneously mixed. An accurately weighed amount of the pulverized tablets equivalent to 100 mg was transferred into a 100-mL VF and subsequently, the active constituent was extracted with 20 mL BDW three times using the sonicator for five minutes each time. The solution developed from the extraction process was filtrated into another 100-mL VF then, the residue was rinsed several times with 1 mL BDW. The solution was totaled to 100 mL using BDW to get (1 mg/mL) as a working solution for conductometric platforms processing.

For the Ag-NPs platform, an accurately weighed quantity of the pulverized tablets equal to 20 milligrams was placed into a 100-mL VF and then, the active constituent was extracted as stated before. The solution resulting from the extraction process was filtrated and handled similarly to that of the conductometric platforms. Afterward, the solution was diluted into a 10-mL VF using BDW to get a working solution of 20 µg/mL for the Ag-NPs platform processing.

Lastly, the analysis was done as displayed under the corresponding general procedure for each proposed platform to estimate the concentration of DACH in its commercial tablets and to carry out the standard addition (SA) technique.

### Application of Ag-NPs platform to spiked human plasma

To a group of centrifugation tubes, 1 mL volumes of thawed human plasma were transferred & subsequently spiked with aliquots of different concentrations (Cs) from DACH standard solution (20 µg/mL). Subsequently, the spiked plasma samples were mixed with methanol (three mLs) for 2 min using the vortex device for protein precipitation. The solutions were centrifuged for twenty minutes at 5000 rpm for the separation of precipitated matrices. The supernatants were cautiously detached and then vaporized to dryness. The dried remains were reconstituted with BDW and directly placed into a group of 10-mL VFs. Afterward, the solutions were treated as displayed under the Ag-NPs platform’s general procedure to get Cs ranging from 600 to 1600 ng/mL. Without DACH, the blank sample was concurrently prepared by the same steps. The concentrated solutions of DACH in plasma were finally estimated from the corresponding regression equation.

### Application of Ag-NPs platform to spiked human urine

The urine samples after thawing at room temperature were diluted tenfold with BDW, centrifuged at 1500 rpm for 1 min, and filtered through 0.45-µm membrane filters. In a series of 10-mL VFs, 1-mL of urine samples were spiked with aliquots of different Cs from DACH standard solution (20 µg/mL) and mixed well for 1 min. Then, the solutions were treated as stated under the Ag-NPs platform’s general procedure to get Cs ranging from 600 to 1600 ng/mL. Without DACH, the blank sample was prepared concomitantly by the same steps. Finally, the concentrated solutions of DACH in urine were estimated from the corresponding regression equation.

### Application of Ag-NPs platform to in-vitro dissolution test

The dissolution experiment was implemented on Daklanork® (66 mg) tablets using the USP dissolution apparatus (type-II; Paddle). This apparatus was set at 75 rpm for 45 min consistent with FDA guidelines [29]. The volume of dissolution media required for test performance was one liter of (5 × 10^− 2^ M) phosphate buffer (pH = 6.8) that was controlled thermostatically at 37 ± 0.5ºC. 10-mL volumes at time intervals of 10, 15, 20, 30, and 45 min were withdrawn, filtered into a group of 25-mL VFs using syringe filters (0.45-µm), and subsequently treated after appropriate dilutions as stated under the Ag-NPs platform’s general procedure. At the time intervals, the withdrawn volumes were substituted with the same volumes of freshly prepared dissolution media. The absorbance values of samples were measured and consequently, the drug release percentage was computed.

## Results and discussion

DACH has a remarkable role not only in the management of moderate or severe COVID-19 pandemic symptoms but also in the therapy of chronic HCV infection according to the previously presented mechanisms. In drug synthesis and/or drug analysis, most labs are embracing green chemistry to diminish negative influences on the environment and to enhance analysts’ health or safety. Hence, four simple green platforms including Ag-NPs technique and three electrochemical conductometric ones were introduced for DACH rapid assay in its tablets, biological fluids, and dissolution media generating excellent recoveries relative to the reported platform’s recoveries [11].

**Fig. 2:**
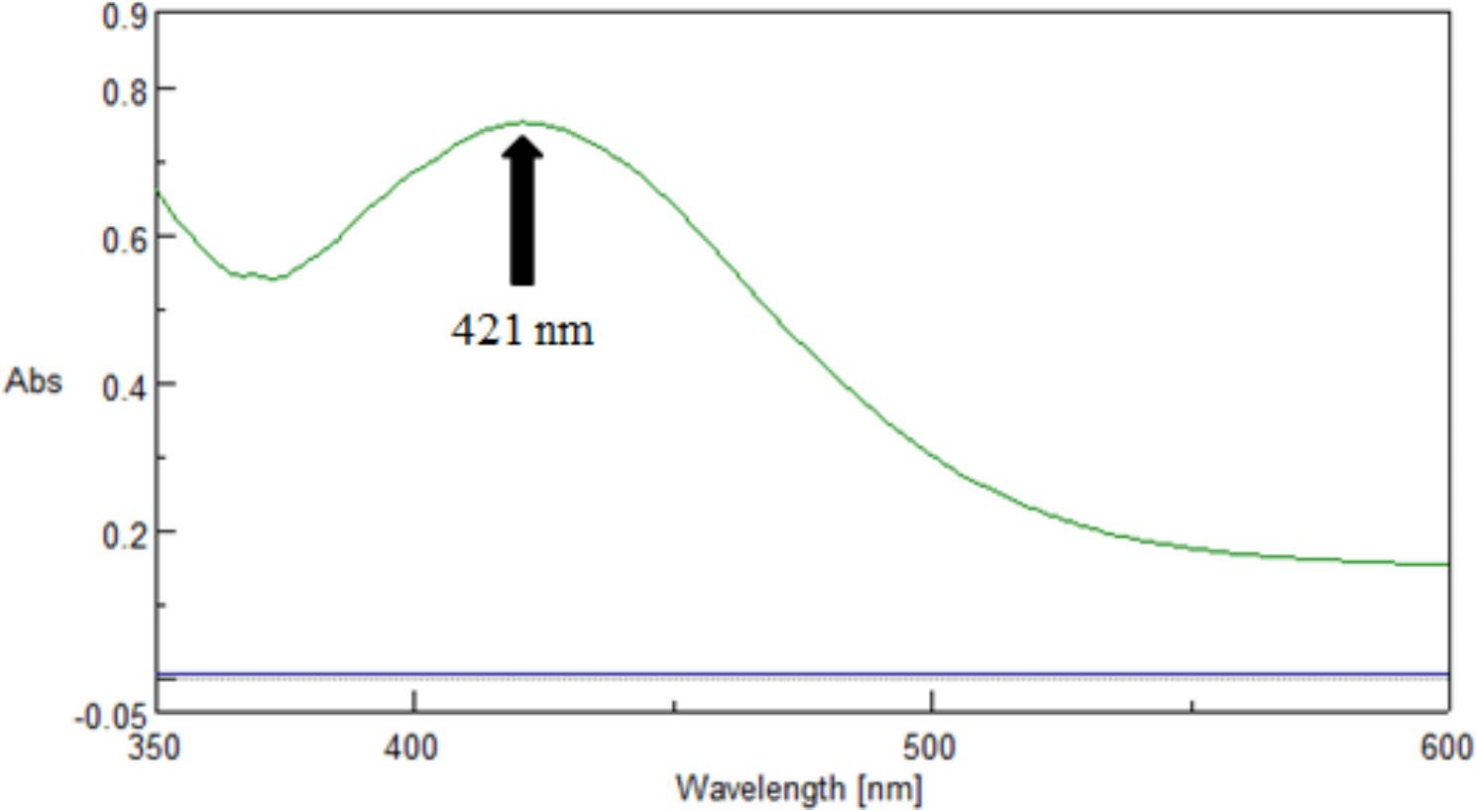
Absorbance spectrum of Ag-NPs developed in presence of DACH (3 µg/mL)

**Fig. 3:**
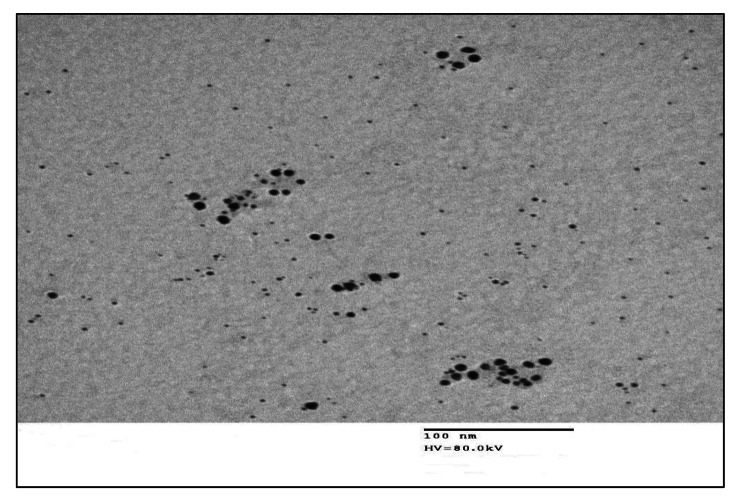
TEM image of Ag-NPs formed in the presence of DACH

For the Ag-NPs platform, the reaction included an AgNO_3_ solution in alkaline media of NaOH with the stabilizer (PVP) to inhibit Ag-NPs’ agglomeration after synthesis or preparation. The addition of DACH to the reaction mix resulted in reduction of all silver ions (Ag^+^) to a stoichiometrically equal mass of Ag-NPs with intriguing optical properties (Figure S1). The Ag-NPs were identified after synthesis by UV-VIS spectrophotometry and transmission electron microscopy. Consequently, the synthesized Ag-NPs displayed a characteristic absorption peak at 421 nm as a result of the surface plasmon excitation (Fig. 2). Also, it was remarked that the DACH absence from this reaction led to the absenteeism of any absorption spectrum from 400 to 700 nm. As exhibited in Fig. 3, the Ag-NPs production in the existence of DACH was checked via the TEM image which disclosed that the synthesized Ag-NPs were spherical with a size of 8.01 ± 1.77 nm and smooth surface morphology. Unlike traditional spectrophotometric platforms, the suggested Ag-NPs platform was highly sensitive to determining very small Cs of DACH and consequently, can be exploited for the pharmacokinetic study of DACH in biological fluids.

**Fig. 4:**
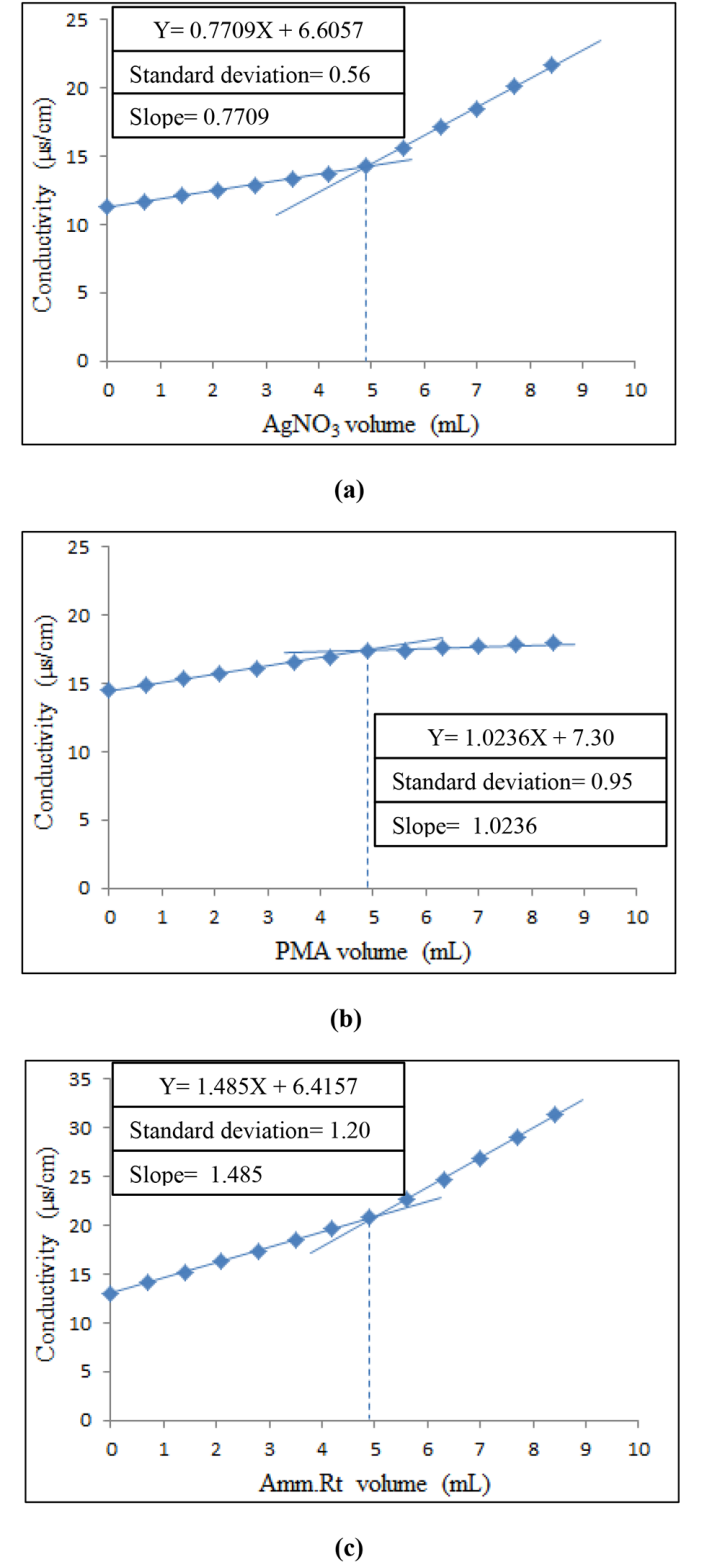
Conductometric titration curves of DACH (10 mg/50mL) versus volumes of (5 × 10^− 3^ M) solutions of: **(a)** AgNO_3_. **(b)** PMA. **(c)** Amm.Rt

For the electrochemical conductometric platforms, the reaction system involved precipitation titrations for quantitative green analysis of DACH by using three proposed reagents (AgNO_3_, PMA, and Amm.Rt). The conductance values of DACH solution varied during the titration process before & after the equivalence point (EP). During titration of DACH solution by the proposed reagents, stable ion pairs with different aqueous solubilities were gradually formed resulting in the first linear part of the titration curve where the conductance values were increased regularly up to the EP. Subsequently, there was an abrupt rise in the conductance values or an abrupt change in the curve’s slope because of the excess volumes of the proposed reagents resulting in the second linear segment of the titration curve. Consequently, the endpoint was graphically computed at the two linear segments’ intersection point and subsequently utilized for computing of DACH amount (Fig. 4a and b, and 4c). For computing the DACH-reagent molar ratio, the same reaction system of the conductometric platforms was applied using equimolar solutions (5 × 10^− 3^ M) of DACH and each proposed reagent. The results proved that the molar ratio of DACH to AgNO_3_ or Amm.Rt was 1:2 while the molar ratio of DACH to PMA was 1:1. Conclusively, the suggested conductometric platforms as eco-friendly electrochemical techniques are appropriate for simple and rapid analysis of DACH in its pharmaceutical at a low cost owing to their reliance only on the BDW (the cheapest and greenest solvent).

## Optimization of Ag-NPs platform variables

To get optimal results of the Ag-NPs platform for the estimation of DACH, the following four variables were examined:

### Effect of AgNO_3_ solution

Numerous experimentations were implemented on differently concentrated AgNO_3_ solutions employing the same concentration of DACH in each experiment under other optimal reaction circumstances. Accordingly, it was noticed that AgNO_3_ solution of 3 × 10^− 3^ M was the best-concentrated solution for optimal outcomes, after which the rise in AgNO_3_ solution’s concentration led to a substantial decline in the absorbance values of prepared Ag-NPs as a result of AgCl (white precipitate) formation. Afterward, various volumes of AgNO_3_ solution of 3 × 10^− 3^ M were tested under the same circumstances of reaction. The outcomes disclosed that one mL volume was the best aliquot for optimal outcomes, after which the Ag-NPs’ absorbance was nearly equal in value with the rise in AgNO_3_ volume (**Figure S2a**).

### Effect of stabilizer

Ag-NPs are prone to agglomerate throughout their production. Therefore, Ag-NPs were steadied or stabilized via electrostatic stabilizers (ESs) or steric stabilizers (SSs) to inhibit their agglomeration [7]. ESs, for example, sodium citrate, work by adsorption on NPs’ surface creating a double electrical layer that triggers columbic repulsions between the NPs and accordingly inhibits their agglomeration. Whereas SSs for example PVP, work by creating a protecting cap on NPs’ surface, consequently inhibiting their agglomeration. Regarding our experiment, it was noted that utilizing PVP stabilizer produced higher absorbances than sodium citrate stabilizer. Therefore, PVP was elected to stabilize the Ag-NPs and inhibit their agglomeration.

Numerous experiments were implemented on differently concentrated PVP solutions in a manner like to that of AgNO_3_. Accordingly, it was noted that PVP (0.14% solution) was the best-concentrated solution for optimal outcomes, after which the rise in PVP concentration (0.14%) resulted in a small decline in the absorbance values of Ag-NPs. Afterward, various volumes of PVP (0.14% solution) were tested under the same circumstances of reaction. The results disclosed that 1.2 mL volume was the best aliquot for optimal outcomes, after which the absorbance values of Ag-NPs diminished a little with the rise in PVP volume (**Figure S2b**).

### Effect of NaOH solution

Throughout the reduction of Ag^+^ to Ag-NPs via DACH, the H^+^ (hydrogen ions) were generated in reaction media. Also, it was noticed that buffer solutions failed to get Ag-NPs. Therefore, NaOH was inserted to grant sufficient alkalinity to the media of reaction & to expend the generated H^+^ ions and consequently hastening the reaction & stimulating the reduction process needed for Ag-NPs production. Accordingly, the NaOH influence should be investigated precisely by trying various concentrated NaOH solutions in a manner like to that of AgNO_3_. After numerous experiments, it was noted that NaOH (5 × 10^− 3^ M) was the best-concentrated solution for optimal outcomes, after which the rise in NaOH concentration led to a substantial decline in the absorbance values of Ag-NPs as a result of Ag_2_O (black precipitate) formation. As well, various aliquots of NaOH (5 × 10^− 3^ M solution) were tried under the same circumstances of reaction. The outcomes disclosed that 1 mL volume was the best aliquot for optimal outcomes, after which the rise in NaOH volume led to a gradual small decline in the absorbance values of Ag-NPs (**Figure S2c**).

### Effect of temperature

It was noted that the Ag-NPs platform’s reaction system necessitated heating at 90 °C for a specific time to get optimal absorbances of Ag-NPs. After this, the rise in the reaction temperature led to a substantial decline in the absorbance values of Ag-NPs owing to silver precipitation. Afterward, various heating times (by minutes) at 90 °C were tried in a manner like to that of the AgNO_3_. It was noted that heating for 30 min was the best time (at 90 °C) for optimal absorbance outcomes, after which the Ag-NPs absorbance persisted constantly representing the end of the reaction to synthesize Ag-NPs (**Figure S2d**).

## Optimization of electrochemical platforms’ variables

To obtain optimum results with higher conductance values and sharp endpoints of the conductometric platforms for the estimation of DACH, the following three variables were investigated:

### Effect of solvent

By testing different solvents such as (BDW, ethanol, 50% v/v ethanol-BDW, methanol, 50% v/v methanol-BDW, acetone, and 50% v/v acetone-BDW) for both DACH and other precipitating reagents, it was observed that aqueous media was the best media for optimum outcomes for the three platforms. Hence, BDW as an eco-friendly solvent was the cheapest and the best media of choice for electrochemical conductometric determination of DACH.

### Effect of reagent concentration

Different prepared Cs of the proposed reagents (AgNO_3_, PMA, and Amm.Rt) were investigated using the same DACH concentration at other optimal reaction circumstances. It was observed that (5 × 10^− 3^ M) of all proposed reagents was the optimum concentration for highly constant and stable conductance readings after well-mixing for two minutes. While unstable conductance values were observed for the Cs of the proposed reagents less than (5 × 10^− 3^ M) and consequently consumed more time to achieve constant readings.

### Effect of temperature

Upon raising the reaction temperature to 40 °C, no alteration was observed in conductance values. Consequently, all conductance experiments were operated at room temperature (25 °C).

## Methods validation

According to ICH guiding principles [30] and the optimized experimental conditions, the suggested green platforms were validated.

### Linearity

The linearity of the Ag-NPs platform was assessed by analyzing ten Cs of DACH (0.50, 1, 1.50, 2, 2.50, 3, 3.50, 4, 4.50, and 5) µg/mL. Also, the calibration graph was created by plotting the absorbances at 421 nm against the matching Cs followed by computing the regression parameters (Table 1). While the linearity and other analytical parameters of the conductometric techniques were assessed by examining seven Cs of DACH (2, 4, 6, 8, 10, 12, and 14) mg/50mL for each proposed reagent yielding satisfactory outcomes (Table 1 and **S1**).

**Table 1:** Assay parameters for the green analysis of DACH by the suggested platforms

Methods/ Parameters	Ag-NPs platform	Conductometric platforms		
		AgNO_3_	PMA	Amm.Rt
Concentration range	0.5-5 (µg/mL)	2–14 (mg/50mL)		
Correlation coefficient	0.9997	0.9996	0.9996	0.9996
Slope	0.2342	0.7709	1.0236	1.485
Intercept	0.0346	6.6057	7.30	6.4157
S.D of intercept*	0.006	0.09	0.12	0.16
S.D of slope	0.002	0.009	0.012	0.018
LOD**	0.09 (µg/mL)	0.385	0.386	0.355
LOQ**	0.27 (µg/mL)	1.167	1.172	1.077

*Standard deviation of intercept

**LOD= (SD of the response/slope) × 3.3; LOQ= (SD of the response/slope) × 10

The regression equation for Ag-NPs platform: Y = 0.2342X + 0.0346.

The regression equation for AgNO_3_ titrant: Y = 0.7709X + 6.6057.

The regression equation for PMA titrant: Y = 1.0236X + 7.30.

The regression equation for Amm.Rt titrant: Y = 1.485X + 6.4157.

### LOD and LOQ for Ag-NPs platform

To estimate the Ag-NPs platform’s sensitivity, we computed LOD & LOQ as displayed in Table 1. The displayed outcomes indicated the high sensitivity of the Ag-NPs platform for DACH estimation.

### Accuracy and precision

To calculate accuracy & precision, 3 standard Cs of DACH were chosen to cover all ranges of the calibration plot (1.5, 3, and 4.5 µg/mL) for the Ag-NPs platform while (4, 8, and 12 mg/50mL) for each proposed reagent of the conductometric platforms and then analyzed (in triplicate) by the suggested green platforms. The accuracy outcomes expressed as (mean ± SD) were excellent for all suggested techniques (**Table S2**). Also, the RSDs% didn’t surpass 2% divulging excellent precision for all platforms as exhibited in **Table S2**, where we also listed the percentage relative errors (Er%) for all methods.

### Robustness for Ag-NPs platform

To evaluate the robustness of Ag-NPs platform, each parameter involved in the reaction was altered individually while maintaining the other variables as-is. The results as displayed in (**Table S3**) disclosed that the Ag-NPs platform wasn’t affected by the intentional changes to the reaction parameters denoting the robustness of this green platform.

## Methods application

### Pharmaceutical application

The proposed green platforms were exploited for quantitative DACH analysis in Daklanork^®^ tablets. The % recoveries’ mean (Found %) & standard deviations (SD) displayed in Table 2 were acceptable and well-aligned with the studied drug’s label claim without intrusion by pharmaceutical additives. Also, Table 2 results have conclusively proven the appropriateness of the green techniques for DACH routine determination in QC labs (the reference range for routine DACH analysis was 4–16 µg/mL). Upon application of the SA technique, satisfactory results were obtained (Table 2), revealing no interference from pharmaceutical additives. Also, the proposed techniques’ validity was tested by analyzing the same samples using our suggested platforms in parallel with the reported platform as presented in **Table S4**, where mean and standard deviation (SD) values were satisfactory.

**Table 2:** Determination of DACH by the suggested platforms in Daklanork® tablets and application of standard addition technique

Methods/ Parameters	Ag-NPs platform	Conductometric platforms		
		AgNO_3_	PMA	Amm.Rt
Daklanork®* Found % ± SD**	99.83 ± 1.16	99.94 ± 0.84	99.63 ± 0.97	99.32 ± 0.55
Standard addition Pure found% ± SD**	100.74 ± 0.90	100.80 ± 0.87	101.28 ± 1.02	100.44 ± 1.20

* Daklanork® tablets labeled to contain 66 mg DACH per tablet; batch number (M171015)

** Mean of five determinations

### Spiked human plasma and urine application

According to DACH’s pharmacokinetic study (PS) [1, 31], the DACH C_max_ within 1 h was 1726 ng/mL after single-dose administration of 60 mg daclatasvir tablet, and about 6.6% of this dose (60 mg) was excreted in urine as daclatasvir. The achieved high sensitivity by the Ag-NPs platform allowed the estimation of DACH at ultra-trace quantities in human plasma and urine. Moreover, the satisfying outcomes exhibited in Table 3 confirmed that the Ag-NPs platform can be exploited in the PS of DACH without interference by matrices of plasma & urine.

**Table 3:** Determination of DACH by the suggested Ag-NPs platform in spiked human plasma and urine

Plasma			Urine		
Added (ng/mL)	Found* (ng/mL)	Recovery%	Added (ng/mL)	Found* (ng/mL)	Recovery%
600	593.65	98.94	600	589.34	98.22
800	785.58	98.20	800	796.28	99.54
1000	992.98	99.30	1000	997.89	99.79
1200	1219.01	101.58	1200	1183.76	98.65
1400	1397.72	99.84	1400	1410.74	100.77
1600	1608.17	100.51	1600	1587.62	99.23
Mean ± SD		99.73 ± 1.20	Mean ± SD		99.37 ± 0.89

* Mean of three determinations

### In-vitro dissolution test application

**Fig. 5:**
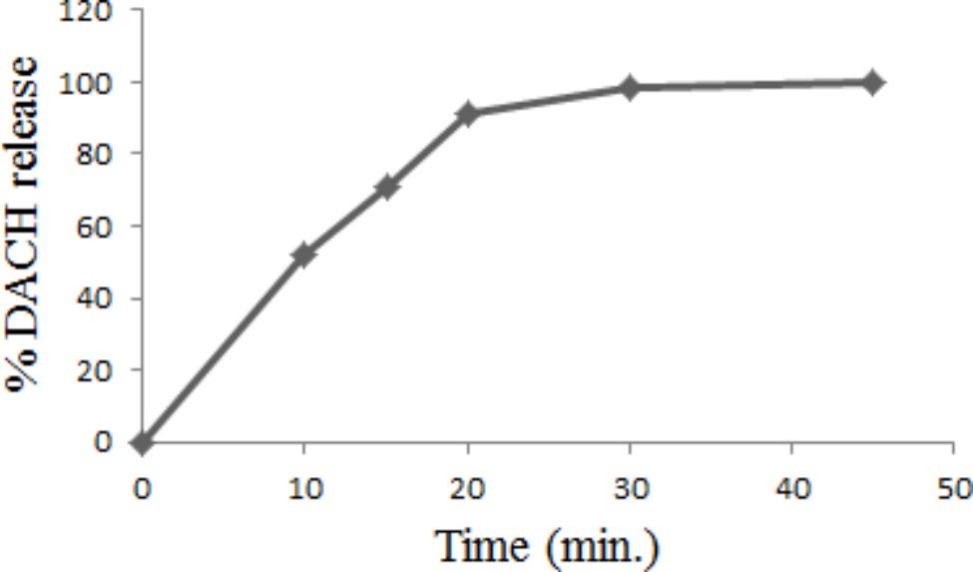
In-vitro dissolution profile of Daklanork® tablet using Ag-NPs platform

The dissolution experiment is crucial for the QC of the marketable product and for foretelling the right delivery of the needed active constituent (DACH) dose to the patients. To evaluate DACH release from the commercial tablets, the dissolution experiment was done on Daklanork^®^ tablets using the Ag-NPs platform. Finally, the percentage of DACH release was estimated using the Ag-NPs platform, then charted vs. the time intervals (Fig. 5).

### Statistical analysis

The results obtained from the suggested green platforms for DACH assay in its pure formula were compared statistically with the reported platform’s outcomes [11]. As well, *t-* & F- values were estimated as in **Table S4**, where the estimated values didn’t outstrip the theoretical values. In line with the statistical comparison (SC) in **Table S4**, insignificant differences were found in the results of the proposed techniques & the reported technique signifying the high accuracy & high precision of the green proposed techniques.

## Conclusion

It could be deduced from the previous discussion that the suggested platforms are simple, specific, precise, and eco-friendly owing to their reliance primarily on water. Because of the attained high accuracy & selectivity by the proposed techniques at the lowest cost, these platforms are considered to be suitable for DACH routine analysis not only in QC labs but also for any future applications with minimal manipulation steps in its pure powder or commercial product. Furthermore, they are considered cheap and green substitutes for chromatographic expensive techniques. Also, the high sensitivity attained by the Ag-NPs platform allowed the estimation of DACH in human plasma & urine. Consequently, the Ag-NPs platform can be exploited in the PS of DACH without interference by matrices of plasma & urine.

## Electronic supplementary material

Below is the link to the electronic supplementary material.

Supplementary Material 1

## Data Availability

All data generated or analyzed during this study are included in this published article [and its supplementary information files].

## Abbreviations

DACH: Daclatasvir dihydrochloride
HCV: Hepatitis C virus
NS5A: HCV protein
Ag-NPs: Silver-nanoparticles
DD: Drug delivery
DA: Drug analysis
AgNO_3_: Silver nitrate
PMA: Phosphomolybdic acid
Amm.Rt: Ammonium reineckate
BDW: Bi-distilled water
PVP: Polyvinylpyrrolidone
K_cell_: Cell Constant
TEM: Transmission electron microscopy
VF: Volumetric flask
SA: Standard addition
Cs: Concentrations
Ag^+^: Silver ions
EP: Equivalence point
ESs: Electrostatic stabilizers
SSs: Steric stabilizers
H^+^: Hydrogen ions
Er%: Percentage relative error
SD: Standard deviation
PS: Pharmacokinetic study
SC: Statistical comparison
