# Supplementary material for: Comparative study of four innovative earth-friendly platforms for rapid analysis of daclatasvir dihydrochloride: Application on different matrices
Source: BMC Chem. 2023 Mar 15;17(1):20. doi: 10.1186/s13065-023-00923-4 (PMC10016150; doi:10.1186/s13065-023-00923-4)
Supplement: Supplementary file 1 — Supplementary Material 1 [file 13065_2023_923_MOESM1_ESM.docx]

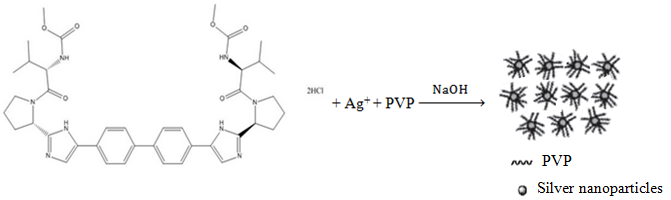

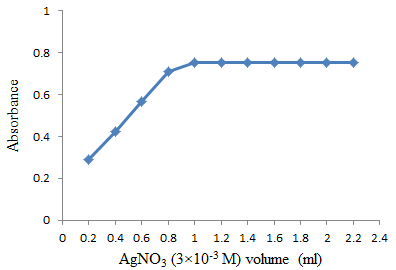
**Figure S1:** Silver ions reduction by DACH to stoichiometrically equivalent quantity of Ag-NPs.

**
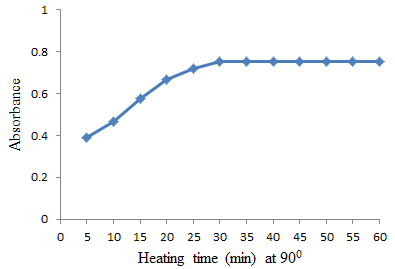

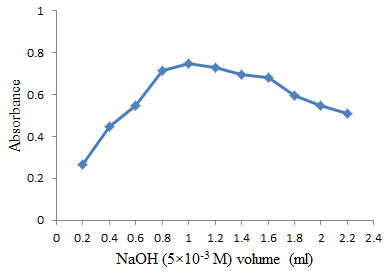
**
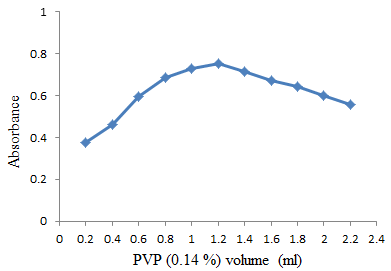
 **(a) (b)**

**(c) (d)**

**Figure S2:** Optimization of Ag-NPs platform variables for DACH (3 μg/mL**)** including: **(a)** Volume of AgNO_3_ (3×10^-3^ M) solution. **(b)** Volume of PVP (0.14%) solution. **(c)** Volume of NaOH (5×10^-3^ M) solution. **(d)** Heating time effect at 90°C.

**Table S1: Electrochemical conductometric analysis of DACH in its pure form using (5×10^-3^ M) of AgNO_3_, PMA, and Amm.Rt.**

| AgNO_3_ | | | PMA | | | Amm.Rt | | |
| --- | --- | --- | --- | --- | --- | --- | --- | --- |
| Added  (mg/50mL) | Found (mg/50mL) | Recovery%* | Added (mg/50mL) | Found (mg/50mL) | Recovery%* | Added  (mg/50mL) | Found (mg/50mL) | Recovery%* |
| 2 | 1.97 | 98.50 | 2 | 2.01 | 100.50 | 2 | 2.03 | 101.50 |
| 4 | 4.03 | 100.75 | 4 | 3.97 | 99.25 | 4 | 3.98 | 99.50 |
| 6 | 5.95 | 99.17 | 6 | 5.93 | 98.83 | 6 | 5.89 | 98.17 |
| 8 | 7.96 | 99.50 | 8 | 7.93 | 99.13 | 8 | 7.97 | 99.63 |
| 10 | 10.04 | 100.40 | 10 | 10.11 | 101.10 | 10 | 9.98 | 99.80 |
| 12 | 11.95 | 99.58 | 12 | 12.07 | 100.58 | 12 | 11.91 | 99.25 |
| 14 | 14.17 | 101.21 | 14 | 13.94 | 99.57 | 14 | 13.90 | 99.29 |
| Mean ± SD | | 99.87  ±  0.95 | Mean ± SD | | 99.85  ±  0.87 | Mean ± SD | | 99.59  ±  0.99 |
| RSD% | | 0.95 | RSD% | | 0.87 | RSD% | | 0.99 |
| SE | | 0.36 | SE | | 0.33 | SE | | 0.37 |
| Variance | | 0.90 | Variance | | 0.76 | Variance | | 0.98 |

* Mean of three determinations. RSD= Relative standard deviation. SE= Standard error.

**Table S2: Intra-day, inter-day precision, and accuracy data obtained for DACH assay by the suggested platforms.**

| Methods  Parameters | Ag-NPs platform | Conductometric platforms | | |
| --- | --- | --- | --- | --- |
|  |  | AgNO_3_ | PMA | Amm.Rt |
| Accuracy |  | | | |
| Mean ± SD | 100.27 ± 1.17 | 100.40 ± 0.72 | 100.17 ± 0.73 | 100.86 ± 0.44 |
| RSD% | 1.17 | 0.72 | 0.73 | 0.44 |
| Er%* | 1.67 | 0.63 | 0.25 | 0.83 |
| Intra-day precision** |  | | | |
| Mean ± SD | 101.67 ± 0.35 | 100.35 ± 1.20 | 100.22 ± 0.28 | 100.88 ± 0.54 |
| RSD% | 0.34 | 1.20 | 0.28 | 0.75 |
| Er%* | 1.77 | 0.13 | 0.50 | 0.50 |
| Inter-day precision*** |  | | | |
| Mean ± SD | 99.51 ± 1.12 | 101.18 ± 0.93 | 100.49 ± 0.52 | 101.85 ± 0.26 |
| RSD% | 1.13 | 0.92 | 0.52 | 0.26 |
| Er%* | -1.11 | 1.38 | 0.75 | 1.75 |

* Relative error percentage.

** The intra-day analysis, average of three different concentrations of DACH (1.5, 3, and 4.5 μg/mL) for the Ag-NPs platform while (4, 8, and 12 mg/50mL) for each proposed reagent of the electrochemical conductometric platforms repeated three times within the day.

*** The inter-day analysis, average of three different concentrations of DACH (1.5, 3, and 4.5 μg/mL) for the Ag-NPs platform while (4, 8, and 12 mg/50mL) for each proposed reagent of the electrochemical conductometric platforms repeated three times in three consecutive days.

**Table S3: Robustness study of the suggested Ag-NPs platform using pure (3 μg/mL) of DACH.**

| Variation | DACH (3 μg/mL) |
| --- | --- |
|  | Recovery%* ± SD |
| No variation  (optimum condition) | 100.40 ± 0.79 |
| AgNO_3_ (3×10^-3^ M) (0.9 mL) | 98.55 ± 0.43 |
| AgNO_3_ (3×10^-3^ M) (1.1 mL) | 100.88 ± 0.26 |
| PVP (0.14%) (1.1 mL) | 98.76 ± 0.16 |
| PVP (0.14%) (1.3 mL) | 98.62 ± 1.09 |
| NaOH (5×10^-3^ M) (0.9 mL) | 99.13 ± 0.64 |
| NaOH (5×10^-3^ M) (1.1 mL) | 99.26 ± 0.99 |
| Heating time (at 90°C) (27 min) | 98.21 ± 0.14 |
| Heating time (at 90°C) (33 min) | 100.67 ± 1.13 |

***** Mean of three determinations.

**Table S4: Statistical comparison between the results obtained by applying the suggested platforms and the reported spectrophotometric method [11] for DACH analysis in its pure form.**

| Methods  Parameters | Ag-NPs platform | Conductometric platforms | | | Reported platform [11] |
| --- | --- | --- | --- | --- | --- |
|  |  | AgNO_3_ | PMA | Amm.Rt |  |
| Mean | 100.27 | 100.40 | 100.17 | 100.86 | 100.01 |
| SD | 1.17 | 0.72 | 0.73 | 0.44 | 1.28 |
| N | 6 | 6 | 6 | 6 | 6 |
| Variance | 1.37 | 0.52 | 0.53 | 0.19 | 1.64 |
| Student^'^s  *t*-test (2.23)* | 0.37 | 0.65 | 0.27 | 1.54 | ------- |
| F- value (5.05)* | 0.84 | 0.32 | 0.33 | 0.12 | ------- |

* The parentheses contain the corresponding theoretical *t* and F values at (*P*=0.05).
